# Monocyte to high-density lipoprotein cholesterol ratio and serum uric acid in Chinese adults: a cross-sectional study

**DOI:** 10.1186/s12902-022-00966-z

**Published:** 2022-02-26

**Authors:** Yuexi Li, Xiaoqin Liu, Yuhan Luo

**Affiliations:** Health Management Center, Deyang People’s Hospital, No. 173, Taishan North Road, Deyang City, Sichuan Province China

**Keywords:** Monocytes, High-density lipoprotein cholesterol, Monocyte to high-density lipoprotein cholesterol ratio, Serum uric acid

## Abstract

**Background:**

Previous studies have shown that the monocyte to high-density lipoprotein cholesterol (HDL-C) ratio (MHR) is a predictor of various diseases such as coronary heart disease, diabetic microangiopathy, and metabolic syndrome. However, there are few scientific reports on the correlation between MHR and serum uric acid. The objective of this report is to explore the relationship between MHR and serum uric acid in Chinese adults.

**Methods:**

This cross-sectional study included 646 participants from southwest China who underwent a health examination at the Health Management Center of Deyang People’s Hospital. The examination included blood pressure readings, routine blood tests (lipid, fasting glucose, serum transaminase, and serum uric acid levels), and various standardized questionnaires. We employed a generalized additive model and smoothed curve fitting to explore the relationship between MHR and serum uric acid levels. We then performed subgroup analyses to investigate the robustness of this relationship.

**Results:**

After adjusting for confounders (age, sex, body mass index, systolic blood pressure, diastolic blood pressure, aspartate transaminase, alanine aminotransferase, fasting glucose, total cholesterol, low-density lipoprotein, smoking, drinking, and exercise status), MHR was found to be positively correlated with serum uric acid levels (*P* < 0.001). The smoothing curve showed an approximately linear correlation between MHR and serum uric acid levels, and the linear correlation coefficient was 146.74 (95% CI 96.16–197.33, *P* < 0.0001). The subgroup analyses showed that the effect of MHR on serum uric acid levels was smaller in occasional smokers and smokers than in nonsmokers (*P* = 0.0194).

**Conclusion:**

MHR was significantly and positively correlated with serum uric acid levels. Additionally, the effect of MHR on serum uric acid levels was lower in the individuals who smoked more.

## Introduction

Hyperuricemia is a dysmetabolic syndrome caused by disruption of purine metabolism. Fluctuations in the human internal environment (e.g., decreased body temperature and pH fluctuations) can decrease the solubility of serum urate, which can then deposit as monosodium urate (MSU) crystals in synovial membranes, kidney medulla, cartilage, and other tissues. MSU accumulation can lead to local inflammation and tissue damage, which eventually leads to the onset of gout or gouty kidney. Hyperuricemia and gout are associated with morbidity and poor prognoses in chronic kidney disease [1–3], cardiovascular disease [2, 4], diabetes [2], atrial fibrillation [5], stroke [6], and dyslipidemia [2]. Monocyte to high-density lipoprotein cholesterol (HDL-C) ratio (MHR), a new predictive marker of inflammation, indicates the ratio of inflammatory markers (monocytes) to anti-inflammatory markers (HDL-C) [7]. MHR is an independent risk factor for metabolic syndrome [8], coronary artery disease [9], and diabetic microangiopathy [10]. MHR might also be associated with the prevalence of hyperuricemia and gout. The main objective of this study is to explore the correlation between MHR and serum uric acid levels in Chinese adults.

## Methods and materials

### Study population

We conducted a cross-sectional study in August 2021 at the Health Management Center of Deyang People’s Hospital, Sichuan Province, China. The study enrolled 646 individuals aged 24–84 years in the southwest of China, according to the following exclusion criteria: 1) presence of an acute gout attack, 2) undergoing uric acid-reducing therapy, 3) presence of acute or chronic infection, 4) presence of abnormal liver/renal function, anemia, bleeding, and hemolytic diseases, 5) presence of uremia, 6) taking medication that might affect hematopoiesis, 7) taking medication that might affect renal function, 8) taking lipid-modulating medication, 9) a history of tumors, and 10) unwillingness to take the questionnaire. This study was approved by the ethics committee of Deyang people’s hospital. All eligible participants were informed of the purpose and process of the study and signed written informed consent before final inclusion. As stated in the declaration of Helsinki, this study follows the principles of biomedical research.

### Clinical and biochemical measurements

After more than 8 h of overnight fasting, the participants had elbow venous blood drawn to test for total cholesterol (TC), HDL-C, low-density lipoprotein cholesterol (LDL-C), glutathione aminotransferase (AST), alanine aminotransferase (ALT), fasting glucose, and serum uric acid, as well as a routine blood examination. Height, weight, systolic blood pressure (SBP) and diastolic blood pressure (DBP) were also recorded. Blood pressure was measured by a designated nurse with a Yueqi arm cylinder electronic sphygmomanometer (Chioy, Beijing, China). Smoking and drinking coffee or strong tea were prohibited for 30 min before the measurement. Participants were asked to empty their bladders and rest quietly for at least 5 min before the measurement. During the measurement, the participants were asked to relax and remain in the seated position without talking. The midpoint of the participants’ upper arm was held at the same level as the heart. The lower edge of the cuff was tied 2.5 cm above their cubital fossa, with appropriate tightness if 1–2 fingers could be inserted between the cuff and participant’s arm. The blood pressure was measured twice at 1–2-min intervals, and the mean of the two measurements was taken as the participant’s final blood pressure. If the difference of the two measurements was greater than 10 mm Hg, a third measurement was taken, and the mean of the last two measurements was taken as the final blood pressure. The participants’ height and weight was measured with a DST-500 ultrasonic measuring instrument (Donghuayuan medical, Beijing, China). During the measurement, participants stood upright on the base plate of the measuring instrument, with their head, heel, sacrum, and two scapular regions in contact with the column. Weight was measured using the gauge’s real-time load cell, while height was measured using the gauge’s ultrasonic probe. The body mass index (BMI) was calculated based on the height and weight [BMI = weight (kg)/ height (m)^2^]. MHR was calculated from the HDL-C level and monocyte count [MHR = monocyte/HDL-C]. The MHR range for this study was 0.062–0.875 µmol/L. Based on the MHR, participants were divided into three groups: low MHR (MHR < 0.227; *n* = 214), middle MHR (0.227 ≤ MHR ≤ 0.345; *n* = 216) and high MHR (MHR > 0.345; *n* = 216).

Based on the standardized methodological recommendations of the World Health Organization (WHO) for smoking surveys [11], we defined smokers as those who smoked more than 1 cigarette a day for at least 6 months continually or accumulatively. Those who smoked more than 4 cigarettes a week but less than 1 cigarette a day were defined as occasional smokers. Individuals who had never or rarely smoked in the past were categorized as nonsmokers, as well as those who smoked daily for at least six months but did not smoke at the time of the survey. To classify drinking status, we considered participants who drank at least once a month as drinkers, those who drank less than once a month but more than once a year as occasional drinkers and the others as nondrinkers [12]. The total intensity of the participants’ various physical activities in the past 7 days was estimated based on the metabolic equivalent (MET) from the International Physical Activity Questionnaire short form survey [13]. Walking refers to walks to work or at home, including walking for transport or exercise, which expends approximately 3.3 METS. Moderate physical activities expend approximately 4.0 METS and refer to those activities that require moderate effort to complete, with slightly deeper respiration than normal (e.g., cycling, ping pong, badminton, and ballroom dancing). Vigorous physical activities expend approximately 8.0 METS and refer to those activities that require significant effort to complete, with significantly deeper respiration than usual (e.g., weight lifting, running, swimming, and prolonged healthy exercise). The total METS (MET-min/week) was calculated as follows: total METS = 3.3 * walking time (min) * number of days that included walking + 4.0 * moderate physical activities time (min) * number of days that included the moderate activity + 8.0 * high-intensity physical activities time (min) * number of days that included the high-intensity activity. Individuals with a total METS ≥ 3000 were assigned to the high-intensity group, those with a total METS of 600–3000 were assigned to the medium-intensity group, and the rest were assigned to the low-intensity group [13].

### Statistical analysis

The continuous variables are expressed as mean ± standard deviation (SD) (normal distribution) or median (maximum, minimum; skewed distribution), and the categorical variables are expressed as percentages. We applied a one-way analysis of variance and Kruskal–Wallis H test to examine the statistical differences in the continuous variables. We used a chi-squared test to examine the statistical differences in the categorical variables. We employed a univariate linear regression model to analyze the association between MHR and serum uric acid levels. The results show the unadjusted, minimally adjusted, and fully adjusted model according to the Strengthening the Reporting of Observational Studies in Epidemiology statement on reporting specifications. Covariates were adjusted or not based on the principle that the matching dominance ratio would be changed by at least 10% after being added to the model [14]. We employed a generalized additive model and smoothed curve fitting to explore the relationship between MHR and serum uric acid levels. If there was a nonlinear relationship, the inflection point of the maximum likelihood model would automatically be calculated with a recursive method [15]. Otherwise, the linear correlation coefficient between MHR and serum uric acid levels would be calculated. Subgroup analyses were performed with a stratified linear regression model. The likelihood ratio test was used to analyze the modifiability and interactions among subgroups. All analyses were performed with the statistical software packages R (http://www.R-project.org, The R Foundation) and EmpowerStats (http://www.empowerstats.com, X&Y Solutions, Inc., Boston, MA). A *p*-value < 0.05 (bilateral) was considered statistically significant.

## Results

### Baseline characteristics of the participants

The participants’ mean age was 49.31 ± 11.16 years, and 57.12% of them were male and 42.88% were female. Table 1 lists the baseline characteristics. There were no statistically significant differences in age and LDL-C levels among the various MHR groups. Compared with the low MHR group, the medium and high MHR groups had significantly higher ALT, AST, BMI, SBP, DBP, fasting glucose, and serum uric acid levels, while the TC level was significantly lower.

**Table 1:** Baseline characteristics of the participants

MHR	Low-level group	Middle-level group	High-level group	*P*-value
Number	214	216	216	
AGE (years, mean ± SD)	49.40 ± 11.11	49.81 ± 10.90	48.70 ± 11.47	0.579
LDL-C (mmol/L, mean ± SD)	2.72 ± 0.70	2.86 ± 0.71	2.80 ± 0.68	0.115
TC (mmol/L, mean ± SD)	4.92 ± 0.90	4.88 ± 0.90	4.71 ± 0.86	0.044
ALT (U/L, mean ± SD)	18.66 ± 10.17	23.77 ± 16.71	30.28 ± 18.17	< 0.001
AST (U/L, mean ± SD)	22.26 ± 6.55	23.63 ± 9.57	25.22 ± 8.77	0.001
BMI (kg/m^2^, mean ± SD)	22.19 ± 2.84	23.77 ± 3.14	25.60 ± 3.03	< 0.001
SBP (mmHg, mean ± SD)	119.64 ± 16.25	122.72 ± 15.51	127.55 ± 16.39	< 0.001
DBP (mmHg, mean ± SD)	71.01 ± 10.87	74.47 ± 10.67	78.00 ± 11.43	< 0.001
Fasting glucose (mmol/L, mean ± SD)	4.87 ± 0.58	5.09 ± 0.80	5.25 ± 1.22	< 0.001
Uric acid (umol/L, mean ± SD)	310.88 ± 72.25	360.71 ± 88.72	404.25 ± 94.84	< 0.001
SEX (n,%)				< 0.001
Male	64 (29.91%)	127 (58.80%)	178 (82.41%)	
Female	150 (70.09%)	89 (41.20%)	38 (17.59%)	
Smoking status (n,%)				< 0.001
Nonsmokers	194 (90.65%)	154 (71.63%)	114 (52.78%)	
Occasional smoking	7 (3.27%)	16 (7.44%)	14 (6.48%)	
Smokers	13 (6.01%)	45 (20.93%)	88 (40.74%)	
Drinking state (n,%)				< 0.001
Nondrinkers	151 (70.56%)	113 (525.6%)	84 (38.89%)	
Occasional drinking	44 (20.56%)	62 (28.84%)	79 (36.57%)	
Drinkers	19 (8.88%)	40 (18.60%)	53 (24.54%)	
Exercise status (n,%)				0.229
Low-intensity group	171 (79.91%)	172 (79.63%)	157 (72.69%)	
Medium-intensity group	29 (13.55%)	25 (11.57%)	33 (15.28%)	
High-intensity group	14 (6.54%)	19 (8.80%)	26 (12.04%)	

*LDL-C* low-density lipoprotein cholesterol, *TC* total cholesterol, *ALT* alanine aminotransferase, *AST* aspartate transaminase, *BMI* body mass index, *SBP* systolic blood pressure, *DBP* diastolic blood pressure

*P* < 0.05

### Univariate analysis

Table 2 lists the results of the univariate analysis showing that MHR, LDL-C, ALT, AST, BMI, SBP, DBP, fasting glucose, smoking, and drinking were positively associated with serum uric acid levels. We also found that age, TC, and exercise were not associated with serum uric acid levels, while the female sex was a protective factor for elevated serum uric acid levels. Compared with the male participants, the female participants’ serum uric acid levels decreased by 103.08 µmol/L on average (*P* < 0.0001).

**Table 2:** Results of the univariate analysis

	Statistics	Effect size (β)	*P*-value
MHR (µmol/L, mean ± SD)	0.31 ± 0.14	285.34 (239.17, 331.51)	< 0.0001
AGE (years, mean ± SD)	49.31 ± 11.16	-0.57 (-1.22, 0.08)	0.0850
LDL-C (mmol/L, mean ± SD)	2.79 ± 0.70	16.92 (6.58, 27.26)	0.0014
TC (mmol/L, mean ± SD)	4.84 ± 0.89	3.68 (-4.46, 11.81)	0.3761
ALT (U/L, median, min–max)	24.26 ± 16.12	1.91 (1.48, 2.33)	< 0.0001
AST (U/L, mean ± SD)	23.71 ± 8.47	2.51 (1.68, 3.35)	< 0.0001
BMI (kg/m^2^, mean ± SD)	23.86 ± 3.31	10.44 (8.41, 12.48)	< 0.0001
SBP (mmHg, mean ± SD)	123.31 ± 16.36	1.07 (0.64, 1.51)	< 0.0001
DBP (mmHg, mean ± SD)	74.50 ± 11.34	2.16 (1.54, 2.78)	< 0.0001
Fasting glucose (mmol/L, mean ± SD)	5.07 ± 0.92	8.93 (1.09, 16.78)	0.0259
SEX (n, %)			
Male	369 (57.12%)	Reference	
Female	277 (42.88%)	-103.08 (-115.35, -90.81)	< 0.0001
Smoking status (n, %)			
Nonsmokers	462 (71.63%)	Reference	
Occasional smoking	37 (5.74%)	55.18 (24.69, 85.66)	0.0004
Smokers	146 (22.64%)	50.93 (33.99, 67.87)	< 0.0001
Drinking state (n, %)			
Nondrinkers	348 (53.95%)	Reference	
Occasional drinking	185 (28.68%)	55.63 (39.88, 71.38)	< 0.0001
Drinkers	112 (17.36%)	75.63 (56.83, 94.43)	< 0.0001
Exercise status (n, %)			
Low-intensity group	500 (77.40%)	0	
Medium-intensity group	87 (13.47%)	15.54 (-5.80, 36.88)	0.1540
High-intensity group	59 (9.13%)	15.61 (-9.68, 40.89)	0.2269

*LDL-C* low-density lipoprotein cholesterol, *TC* total cholesterol, *ALT* alanine aminotransferase, *AST* aspartate transaminase, *BMI* body mass index, *SBP* systolic blood pressure, *DBP* diastolic blood pressure

*P* < 0.05

### The relationship between MHR and serum uric acid

To further demonstrate that MHR was an independent predictor of serum uric acid elevation, we performed the unadjusted and adjusted models with logistic regression analyses. As shown in Table 3, there was a positive correlation between MHR and serum uric acid levels in the unadjusted model (β = 285.340, 95% confidence interval (CI) 239.175–331.506, *P* < 0.0001). There was also a positive correlation in the minimally adjusted model (adjusted for age and sex, β = 155.848, 95% CI 109.641–202.055, *P* < 0.0001) and fully adjusted model (adjusted for sex, age, LDL-C, TC, ALT, AST, BMI, SBP, DBP, fasting glucose, smoking, drinking, exercise status, β = 128.77, 95% CI 79.96–177.59, *P* < 0.0001). We found significantly higher serum uric acid levels in the middle and high MHR groups compared with the low MHR group among all the unadjusted and adjusted models (*P* for the trend < 0.001).

**Table 3:** Relationship between MHR and serum uric acid levels in the various models

	Non-adjusted (β, 95%CI, *P*)	minimally adjusted (β, 95%CI, *P*)	fully adjusted (β, 95%CI, *P*)
MHR (µmol/L)	285.340(239.175,331.506) < 0.0001	155.848(109.641,202.055) < 0.0001	128.77 (79.96, 177.59) < 0.0001
MHR grouping			
Low-level	Reference	Reference	Reference
Middle-level	49.830 (33.603, 66.057) < 0.0001	25.243 (10.240, 40.246) 0.0010	20.51 (5.51, 35.50) 0.0075
High-level	93.371 (77.145, 109.598) < 0.0001	48.003 (31.911, 64.096) < 0.0001	36.33 (19.42, 53.24) < 0.0001
* P* for trend	< 0.001	< 0.001	< 0.0001

Non-adjusted (other covariates were not adjusted)

Minimally adjusted model (adjusted for sex and age)

Fully adjusted model (adjusted for: sex, age, LDL-C, TC, ALT, AST, BMI, SBP, DBP, fasting glucose, smoking and drinking status, and exercise status)

*P* < 0.05

### The linear relationship analyses

**Fig. 1:**
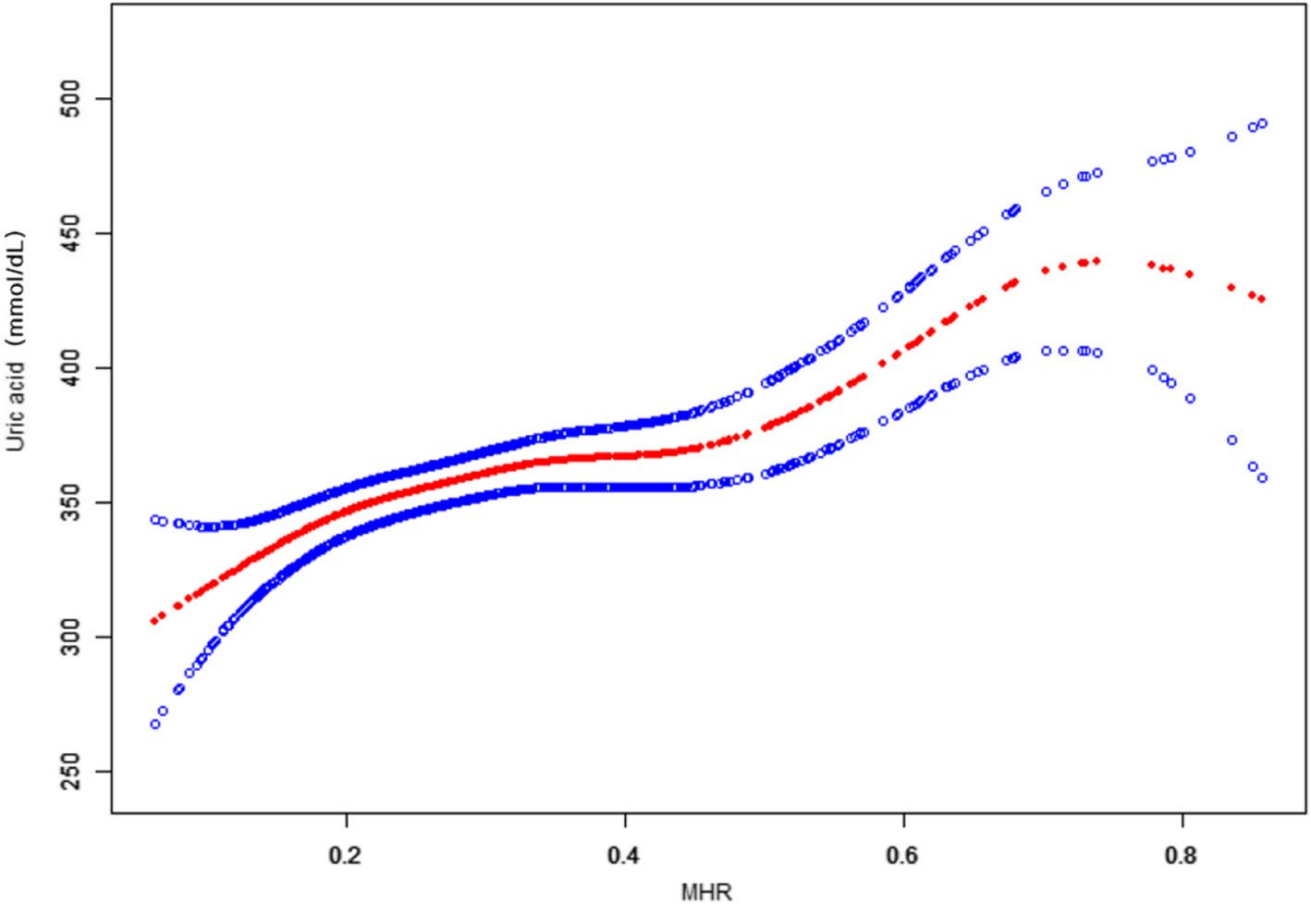
The linear relationship between MHR and serum uric acid levels. A linear relationship was detected after adjusting for sex, age, LDL-C, TC, ALT, AST, BMI, SBP, DBP, fasting glucose, smoking, drinking, and exercise status

After adjusting for covariates, the smooth fitting showed that there was an approximately linear relationship between MHR and serum uric acid levels. As shown in Fig. 1, after adjusting for sex, age, LDL-C, TC, ALT, AST, BMI, SBP, DBP, fasting glucose, smoking, drinking, and exercise status, the linear correlation coefficient of MHR on serum uric acid levels was 146.74 (95% CI 96.16–197.33, *P* < 0.0001) (Table 4).

**Table 4:** Analysis of the concentration-effect relationship

Independent variable	Effect size (β)	95% CI	*P*-value
MHR	146.74	96.16 to 197.33	< 0.0001

Effect: uric acid Cause: MHR

Adjusted for sex, age, LDL-C, TC, ALT, AST, BMI, SBP, DBP, fasting glucose, smoking status, drinking status, exercise status

*P* < 0.05

### The results of the subgroup analyses

As shown in Table 5, the interactive test for smoking status was statistically significant (*P* = 0.0194). For each SD increase in MHR, the serum uric acid levels rose by a mean of 110.9 µmol/L less in the occasional smokers and 134.1 µmol/L less in smokers, compared with the nonsmokers. There were no statistically significant differences in the interactive tests for sex, age, BMI, SBP, DBP, drinking, and exercise status (*P* = 0.5992, *P* = 0.6114, *P* = 0.6586, *P* = 0.5773, *P* = 0.0782, *P* = 0.4333, and *P* = 0.2961, respectively).

**Table 5:** Effect size of MHR on serum uric acid in the established and exploratory subgroups

Characteristic	No of participants	Effect size (95%CI)	*P* (interaction)
SEX			0.5992
male	369	139.6 (82.2, 197.0)	
female	277	167.0 (75.0, 259.1)	
Age (year)			0.6114
≤ 50	349	156.96 (93.43, 220.49)	
> 50	297	135.62 (67.40, 203.83)	
BMI (kg/m^2^)			0.6586
≤ 18.5	27	54.5 (-379.3, 488.3)	
18.5 < BMI ≤ 24	312	174.8 (104.0, 245.7)	
> 24	307	139.5 (75.4, 203.7)	
SBP (mmHg)			0.5773
≤ 120	321	160.7 (89.4, 232.0)	
> 129	325	137.0 (75.9, 198.1)	
DBP (mmHg)			0.0782
≤ 80	452	174.7 (115.1, 234.2)	
> 80	194	96.1 (19.9, 172.3)	
Smoking status			0.0194
Nonsmokers	462	202.6 (138.5, 266.6)	
Occasional smoking	37	91.7 (-87.3, 270.7)	
Smokers	146	68.5 (-10.7, 147.8)	
Drinking status			0.4333
Nondrinkers	348	152.5 (80.4, 224.6)	
Occasional drinking	185	175.6 (97.4, 253.7)	
Drinkers	112	98.7 (4.5, 192.9	
Exercise status			0.2961
Low-intensity group	500	149.7 (92.9, 206.5)	
Medium-intensity group	87	197.2 (87.6, 306.9)	
High-intensity group	59	171.5 (-50.8, 193.9)	

Adjusted for sex, age, LDL-C, TC, ALT, AST, BMI, SBP, DBP, fasting glucose, smoking status, drinking status, and exercise status, but not the stratification variable

*P* < 0.05

## Discussion

The present study sought to explore the relationship between MHR and serum uric acid. Among all the unadjusted and adjusted models, MHR was positively correlated with serum uric acid levels. After adjusting for the covariates, there was an approximately linear relationship between MHR and serum uric acid levels in the smoothed curve, with a correlation coefficient of 146.74 (95% CI 96.16–197.33, *P* < 0.0001), indicating that serum uric acid levels increased by a mean of 146.74 µmol/L for each SD increase in MHR. In addition, the univariate analysis showed that serum uric acid levels in the women decreased by a mean of 103.08 µmol/L compared with the men. This observation is similar to the findings of other studies [16, 17]. The lower serum uric acid levels in the women might be related to estrogen, given that estrogen has been suggested to reduce the protein levels of uric acid reabsorptive transporters, such as uric acid transporter 1, glucose transporter 9, and uric acid efflux transporter ATP-binding cassette subfamily G member 2 [18].

We performed a PubMed search with the keywords “serum uric acid” and “monocyte to high-density lipoprotein cholesterol (HDL-C) ratio” simultaneously. By the end of October 2021, only one scientific paper was found in the database. The finding of a linear correlation between MHR and serum uric acid levels in this study is consistent with the results of Chen et al. based on the Northeast China Rural Cardiovascular Health Study’s (NCRCHS) cross-sectional epidemiological survey [19]; the detailed design and rationale of NCRCHS have been fully described elsewhere [20]. After adjusting for confounders, the authors employed multivariate logistic regression to demonstrate the independent relationship between MHR and the prevalence of hyperuricemia. The authors then used a smoothed curve fitting and a generalized additive model to further describe the linear relationship. Given that stratification analyses are extremely important in scientific research, Chen et al. also performed stratification analyses to detect the robustness of this association. In their study, the stratification variables included age, sex, BMI, SBP, fasting glucose, and estimated glomerular filtration rate. Nevertheless, this adjustment is still controversial. For example, HDL-C has been used to calculate MHR and might therefore not need adjusting as a confounder. LDL-C, ALT, AST, and DBP also affect serum uric acid levels [19, 21, 22] and also need to be matched. To obtain a more complete analysis of the modifiability and interactions, stratification variables such as smoking, drinking, and exercise status should be included in the subgroup analyses. Therefore, the authors’ conclusions are limited. In our study, we used sex, age, BMI, SBP, DBP, smoking, drinking, and exercise status as stratified variables. It is interesting to note that the effect size of MHR on serum uric acid levels significantly differed across the various smoking statuses. For every SD increase in MHR, serum uric acid levels increased by 110.9 mmol/L less in the occasional smokers and 134.1 mmol/L less in the smokers, compared with the nonsmokers.

Monocytes can elevate serum uric acid levels through a variety of pathways. First, monocytes promote the production of inflammatory cytokines such as tumor necrosis factor-alpha (TNF-α), interleukin (IL-6), IL-1β, IL-12, and IL-23 [23] and reduce the levels of anti-inflammatory cytokines (IL-10) [24]. TNF-α can not only damage vascular endothelial cells directly but also induce hyperinsulinemia. Elevated insulin and insulin precursors can stimulate the sodium-hydrogen exchange of renal tubules and increase uric acid reabsorption along with increased hydrogen excretion. Second, the translocation of glycolytic intermediates to ribose 5-phosphate and ribose phosphate pyrophosphate can lead to insulin resistance. Insulin resistance can excite the sympathetic-adrenal medullary system and increase the secretion of catecholamines, which can increase uric acid levels both by increasing the conversion rate of purine bases and inhibiting uric acid excretion. Increased serum uric acid can in turn stimulate the release of inflammatory factors (TNF-α, IL-6) from monocytes, and these inflammatory factors in return lead to an eventual cascade increase in serum uric acid levels [25]. TNF-α can also cause insulin resistance by damaging pancreatic P cells, thereby increasing serum uric acid levels [26]. In contrast, HDL, an anti-inflammatory factor, can reduce the production of inflammatory cytokines and affects a range of immune cell responses and can inhibit the activation, adhesion, and migration of monocytes [27, 28]. Apolipoprotein A-1, the major protein component of HDL-C [25], exerts an inhibitory effect on inflammatory cytokines produced by monocytes by reducing the activation of CD11b. Thus, HDL-C is a protective factor for elevated serum uric acid. All evidence suggests that MHR is positively related to serum uric acid levels. Clearly, the risks factors for hyperuricemia and gout include the male sex [29], age [30], BMI [2, 31, 32], blood pressure [32, 33], alcohol consumption [34, 35], and physical activity [36].

There is as yet no consensus on the relationship between smoking and the prevalence of hyperuricemia and gout [37]. Although numerous studies have demonstrated either a positive or non-significant correlation between smoking and serum uric acid levels [38–40], other studies have also suggested that smoking can lower serum uric acid levels [41–45]. Masahiko Tsuchiya [46] measured concentrations of plasma antioxidants such as uric acid, ascorbic acid, nitrate, cysteine, and methionine in smokers and found that these were significantly lower in the smokers one hour after smoking, suggesting that the free radical component of cigarettes might deplete serum antioxidants (including serum uric acid) [47]. This might partly explain the smaller effect MHR has on serum uric acid in the higher smoking subgroups in our study. Another possible mechanism is that the cyanide in cigarette smoke reduces serum urate by inhibiting xanthine oxidase, a key enzyme of uric acid [48].

Our research has a number of advantages. First, to avoid the potential confounding of an observational study, we not only verified the linear relationship between MHR and serum uric acid levels but also performed rigorous statistical adjustments for sex, age, LDL-C, TC, ALT, AST, BMI, SBP, DBP, fasting glucose, smoking, drinking, and exercise status. Second, the effect modification factor analysis allows for fuller use of the data. The subgroup analyses showed that the effect of MHR on serum uric acid levels was smaller in the occasional smokers and smokers compared with the nonsmokers.

Our study also had certain limitations. First, the study is an analytical cross-sectional study, which provides weak evidence between exposure and results, making it difficult to distinguish causality. Second, the findings might not be generalizable to other ethnic groups due to the fact that the study population included only Chinese participants from the southwest of China.

## Conclusion

MHR was significantly and positively correlated with serum uric acid. In addition, the effect of MHR on serum uric acid levels was lower in the individuals who smoked more.

## Data Availability

The datasets generated and analyzed during the current study are available from the corresponding author upon reasonable request.

## Abbreviations

HDL-C: High-density lipoprotein cholesterol
MHR: Monocyte to high-density lipoprotein cholesterol ratio
MSU: Monosodium urate
TC: Total cholesterol
LDL-C: Low-density lipoprotein cholesterol
AST: Glutathione aminotransferase
ALT: Alanine aminotransferase
SBP: Systolic blood pressure
DBP: Diastolic blood pressure
BMI: Body mass index
WHO: World Health Organization
MET: Metabolic equivalent
SD: Standard deviation
CI: Confidence interval
NCRCHS: Northeast China Rural Cardiovascular Health Study
TNF-a: Tumor necrosis factor-a
IL-6: Interleukin-6
